# Towards faster plan adaptation for proton arc therapy using initial treatment plan information^☆^

**DOI:** 10.1016/j.phro.2025.100705

**Published:** 2025-01-30

**Authors:** Benjamin Roberfroid, Margerie Huet-Dastarac, Elena Borderías-Villarroel, Rodin Koffeing, John A. Lee, Ana M. Barragán-Montero, Edmond Sterpin

**Affiliations:** a Université catholique de Louvain – Center of Molecular Imaging, Radiotherapy and Oncology (MIRO) Brussels Belgium; b KU Leuven – Department of Oncology Laboratory of Experimental Radiotherapy Leuven Belgium; c Particle Therapy Interuniversity Center Leuven – PARTICLE Leuven Belgium

## Abstract

**Background and Purpose:**

Proton arc therapy (PAT) is an emerging modality delivering continuously rotating proton beams. Current PAT planning approaches are time-consuming, making them unsuitable for online adaptation. This study proposes an accelerated workflow for adapting PAT plans.

**Materials and Methods:**

The proposed workflow transfers spots from initial computed tomography (CT) to the CT of the day, updates energy layers considering the initial pattern, and re-optimizes selected transferred spots based on their initial weights and impact on the objective function.

A retrospective study was conducted on five head and neck patients who underwent plan adaptation on a repeated CT. PAT plans were generated with two different methods on the repeated CT: *reference*, created de novo, and *smart-adapted*, generated with the proposed adaptive workflow. Robust optimization was performed for all plans.

**Results:**

*Smart-adapted* plans achieved similar mean dose to organs at risk as the *reference*: the largest median increase of mean dose was 1.9 Gy to the mandible; the median of maximum dose to spinal cord was 0.5 Gy lower for the *smart-adapted* plans. The median target coverage, i.e. D_98_, to primary tumor and nodes of *smart-adapted* plans decreased by 0.2 and 0.4 Gy for the nominal case, and 0.4 and 0.6 Gy for the worst-case scenario; all *smart-adapted* plans met clinical objectives. The smart-adaptation method reduced average planning time from 19184 s to 5626 s, a 3.4-fold improvement.

**Conclusions:**

*Smart-adapted* plans achieve similar plan quality to the reference method, while significantly reducing plan generation time for new patient anatomy.

## Highlights

• Proposed arc energy layer pattern preservation did not impair plan quality.
• Partial spot reoptimization yields similar plan quality to full reoptimization.
• Proposed plan adaptation was 3.4 times faster than conventional full reoptimization.

## 1 Introduction

Proton arc therapy (PAT) is an emerging cancer treatment modality that aims at delivering a proton beam in a rotational pattern around the patient, in contrast to the traditional intensity modulated proton therapy (IMPT), which delivers beams at fixed angles. Conceptually, PAT has more degrees of freedom than IMPT and has thus the potential to achieve better dose distributions [1], [2], [3], [4], [5]. However, several challenges remain to be addressed to harness its full potential.

Like IMPT, PAT remains sensitive to treatment delivery uncertainties and anatomical changes [6]. While robust optimization is commonly used during plan optimization [7], [8] to account for these, it entails larger volumes irradiated at higher doses and may not cover all possible anatomical changes. Given this limitation, proton online adaptation (OA) has until now promised to be the best option to reduce treatment uncertainties while improving dose conformity [9], [10], [11]. Enabling OA for proton therapy is thus often considered as a primordial objective to realize the full potential of this treatment modality.

Unfortunately, the challenge for PAT is to determine optimal energy layers per beam angle. This is needed to achieve optimal dosimetric quality while maintaining a reasonable treatment time [12], [13]. Excessive energy switches, particularly switch-ups (those are much slower due to magnetic hysteresis effect in the delivery system), might indeed result in significantly prolonged treatment delivery that would hardly be compatible with clinical conditions. Different research groups have tackled the problem from different perspectives, resulting in various optimization approaches [12], [14], [15], [16], [17], [18], [19]. These methods exhibit differences in computation times, final energy layer patterns, and dose distribution. Although some methods are faster than others, total PAT optimization times (including the energy layer selection process) are generally slower than conventional IMPT, thus representing a significant obstacle for OA.

In this context, implementing a dedicated online adaptive workflow for PAT, by speeding up plan re-optimization, would ensure that PAT would not lag behind IMPT for situations requiring OA. Currently for IMPT, several solutions have already been proposed by different research groups. Some examples include making use of analytical algorithms to speed up optimization of the dose on the computed tomography (CT) of the day [20], [21], bypassing the manual plan optimization loop by restoring the dose distribution of initial CT onto the CT of the day [22], [23], [24], or mimicking a dose predicted by artificial intelligence (AI) on the CT of the day [11]. Alternatively, Botas et al. proposed a workflow where they re-used the initial spot grid onto the image of the day through a deformation field between both images, followed by the tuning of a subset of these spot weights. Eventually, they managed to adapt IMPT plans in a time span compatible with clinical conditions [25]. Our proposed PAT adaption workflow re-uses the key elements of this workflow while considering the specific challenges of PAT.

This study aims to propose a time-efficient OA workflow for PAT that effectively addresses uncertainties and anatomical changes detrimental to proton therapy, enhancing the clinical quality of treatment plans and ensuring a promising future for the PAT treatment modality.

## 2 Materials and methods

### 2.1 Patient data for workflow evaluation

The study has been retrospectively conducted on five head and neck cancer patients with nodal involvement. The dose prescription was 70 Gy to the clinical target volume of primary tumor (CTV_p_) and 54.25 Gy to CTV of the nodes (CTV_n_), except for the 5th case where a part of the nodal target also receives 70 Gy (CTV_n,high_). CT images were acquired on an Acquilion CT scanner (Toshiba Medical Systems Corporation, Japan) with a slice thickness of 2 mm. For each of these patients, an initial CT (CT_1_) was acquired before treatment and a second CT (CT_2_) was acquired several days after treatment started. In each case, CT_2_ was acquired due to observation of important anatomical changes regarding CT_1_. Organ at risks (OARs) and target contours were segmented by the physician in charge for both CT_1_ and CT_2_. Local ethics committee approval by the CEHF (Comité d'Ethique hospitalo-facultaire) was obtained for the use of all patient data under the agreement “Learning from the past: the MIRO treatment planning database” (2019/16SEP/402).

### 2.2 Adaptive workflow

**Fig. 1:**
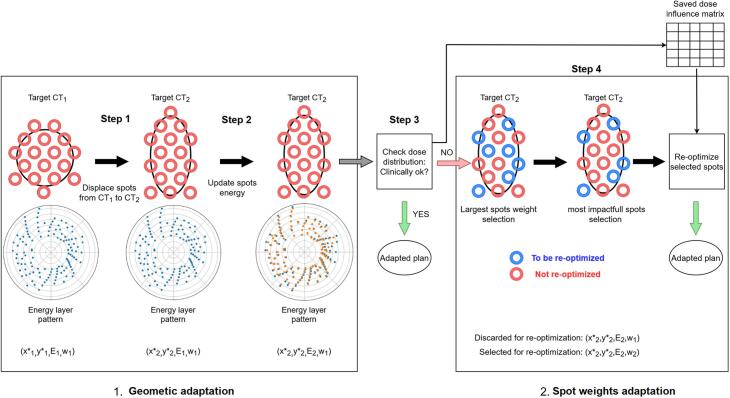
The proposed "smart" PAT adaptation workflow. It consists in two different steps: geometric and spot weights. The first step of the geometric adaptation is to displace the initial spots onto the CT of the day; the second is to update the spot energy while ensuring PAT energy layer pattern. At step three, the dose distribution is verified, if its quality is deemed clinical then the adaptation workflow can already stop there. Otherwise, the dose influence matrix resulting from the dose computation is saved for the step 4. The first step of the spot weights adaptation is to select the largest weights for the re-optimization; the second is to refine this first selection by selecting the spots with the largest derivative of the objective function according to spot weights. Eventually, the weights of the selected spots are re-optimized, using the saved dose influence matrix. Black circular shape on the top denotes a schematic representation of the target volume in 2D, the rings denote a schematic representation of the treatment spots for such target. In the middle, dots disposed on a polar plot illustrate a representation of the energy layers for each beam angle, with the radial distance being the energy and the angular position being the beam angle. The blue dots are the initial energy layers while the orange dots are the updated ones. In the bottom, the quadruplet (x*, y*, E, w) summarizes the state of the spots at each steps. x* and y* denote the spots position in Beam-eye-view, E denotes the energy of the spots, w denotes the spots weight. Subscript "1" indicates that the variable preserves its CT_1_ value, subscript "2" indicates that the variable is updated for CT_2_. (For interpretation of the references to colour in this figure legend, the reader is referred to the web version of this article.)

The PAT adaptive workflow comprises 4 steps. Steps 1–3 form the geometrical adaptation (left of Fig. 1) while Step 4 handles spot weights adaptation (right of Fig. 1). Implementation was done in OpenTPS, a Python-based open-source treatment planning system (TPS) [26].

*Step 1* involves registering the planning CT and spot grid to daily anatomy. After deformably registering CT_1_ to CT_2_, the resulting displacement field transfers spots from CT_1_ to CT_2_. To do so, Bragg peak positions on CT_1_ are retrieved and used as surrogate for the spatial position of the *i* spots (x_i,1_, y_i,1_, z_i,1_) in DICOM (patient-system) coordinates. The displacement field then maps these positions onto CT_2_, i.e., (x_i,2_, y_i,2_, z_i,2_). We used a hexagonal spot grid, and the deformable intensity-based deformable registration tool of the Raystation TPS (Raysearch laboratories, Sweden). The deformation field was then exported to OpenTPS to operate the displacements.

*Step 2* converts updated spot positions (x_i,2_, y_i,2_, z_i,2_) to beam-eye-view coordinates (x*_i,2_, y*_i,2_, E_i,2_), where x* and y* represent horizontal and vertical positions, and E is the necessary energy to reach the desired depth in patient. The determination of the energies can be achieved by a raytracing process on the new anatomy. However, PAT treatments require careful energy assignment to maintain treatment efficiency and quality. Unfortunately, spot registration between CT_1_ and CT_2_ typically disrupts initial spot clusters (spots sharing the same energy layer and beam angle on CT_1_), as this clustering is not considered during the registration process. Consequently, our method proposes to preserve the clusters by updating the energy on CT_2_ while considering the energy layer pattern of CT_1_, i.e., spot clusters are preserved and angles where energy switch-ups occur on CT_1_ remain the same on CT_2_. To do so, for each initial cluster *c* the median energy of updated spots $\tilde{E_{c}}$ is computed and forced to be assigned to the spots of the considered cluster. If for a given angle, $\tilde{E_{c}}$ value leads to a non-authorized energy switch-up (i.e., an increase of energy between two consecutive angles that is not occurring on the initial energy pattern), then the energy for this specific cluster *c* is instead set at the same energy level than the previous cluster, i.e., $\tilde{E_{c}}=\tilde{E_{c-1}}$. We hypothesized this energy layer preservation would maintain the plan quality while keeping beam parameters similar to the initial plan, facilitating PAT plan adaptation.

*Step 3* verifies the dose distribution. The dose is computed in CT_2_ with the updated position and energy of the spots as well as their weights from CT_1_ plan. At this step, if the dose is deemed clinically acceptable, the adaptive process can be stopped. Otherwise, the dose contribution of each spot *i* to the total dose (i.e. the so-called beamlets or dose-influences *D_i_*) are then saved for the following step. These *D_i_* are each generated using proton numbers proportional to their CT_1_ weights. The total dose was computed using MCsquare, an open-source Monte-Carlo engine [27] integrated in OpenTPS, with 3*10^7^ protons.

*Step 4* implements spot weights re-optimization through a two-criteria selection process. First, spots with largest weight (on CT_1_) are selected, i.e., the smallest set of spots carrying at least a percentage *p* of the sum of the spots weight is selected. Spots with small initial weights result in beamlets with high dose uncertainty (i.e. they were generated at step 3 with a low number of protons), which are therefore not suitable for weight re-optimization. The second criterion selects spots with substantial impact on the objective function among those meeting the first criterion, using a gradient constraint.

(1) $$\frac{\partial F}{\partial w_{i}}>t$$

where F is the objective function of the optimization problem, w_i_ is the *i* spot weight and *t* is an arbitrary chosen threshold. Note that discarded spots are not deleted, they still contribute to the total dose distribution similarly to a background dose:

(2) $$\mathrm{TD}=d_{d}+d_{s}=w_{d,1}D_{d}+w_{s,updated}D_{s\;}$$

with *TD* being the treatment dose, *d_d_* the dose contribution from the discarded spots, *d_s_* the dose contribution from selected spots, w_d,1_ the spots weights of discarded spots (thus equal to those on CT_1_ plan), D_d_ the dose influences matrix of the discarded spots, w_s,updated_ the spots weight of selected spots that were re-optimized, and D_s_ the dose influences matrix of the selected spots. For our five patients, we selected spots carrying at least 65 % of total weight (first criterion) and used t = 3 for the gradient constraint (second criterion). These values were determined empirically. While Botas' paper used a 50 % weight threshold, we increased it to 65 % and added the gradient constraint for dosimetric reasons (see supplementary materials).

### 2.3 Plan parameters

Each plan featured a single 360-degree arc with control points at each degree. Energy layers were defined with the ELSA algorithm [4] integrated in the RayStation TPS version 11B. Initial optimization constraints were applied to CT_2_ plans and adjusted when needed. Optimization parameters varied per patient, with constraints and priority weights determined case-by-case. Each adapted plan allowed 500 maximum iterations. All plans incorporated worst-case robust optimization using 2.6 % range error and [4.0,4.0,4.0] mm setup error, based on previous studies [28], [29]. The robust constraints concerned the maximal and minimal dose to the CTV_p_, CTV_n_, CTV_n,high_ as well as the max dose to the spinal cord (SC). Physicians in charge provided contours for both CT_1_ and CT_2_.

### 2.4 Plan comparison

PAT plans were generated for CT_1_ and CT_2_. Plans on CT_2_ were generated with two approaches: “*reference*”, optimized from the ground up; and “*smart-adapted*” generated following the proposed adaptive workflow. The reference plans used the same plan parameters as the smart-adapted plans, except that the spots and energy layers were initialized from the ground up and the dose influences matrices were generated with 10^4^ protons for each spot.

To evaluate the proposed adaptive workflow, the plan quality for the geometric adaptation, i.e., adaption of the spot position and energy only (left box in Fig. 1), on critical constraints was first investigated: D_98__%_ CTV_p_, D_98__%_ CTV_n_, and D_max_ on SC on nominal case, for the reference, the smart-adapted and the no-adapted plan computed on CT_2_ were reported. For these critical clinical goals, we aimed to achieve D_98__%_ > 95 % of the prescribed dose (66.5 Gy and 51.54 Gy for CTV_p_ and CTV_n_ respectively) and D_max_ < 44 Gy to SC. These metrics were evaluated following complete plan adaptation (comprising both geometric adaptation and spot weight refinement) (both boxes in Fig. 1), on the nominal case and the worst-case scenario. Mean dose to OARs was also reported on the nominal case. For other objectives, the reference plans aimed at achieving the lowest mean dose to OARs and the lowest D_2_ for CTV_p_.

A timing comparison between smart-adaptation and reference methods is also provided. Spots and energy initialization, computation time to determine the dose influence matrix, and dose optimization time were reported.

### 2.5 Hardware

The registration process used a computer with an Intel Xeon Gold 6244, 96 GB RAM, and Nvidia RTX A600 GPU. Dose influence matrix generation, adaptive workflow, and optimization used a system with Tesla A100 80 GB GPU, 128 Intel Xeon Gold CPUs, and 125 GB RAM.

## 3 Results

### 3.1 Geometrical adaption

**Fig. 2:**
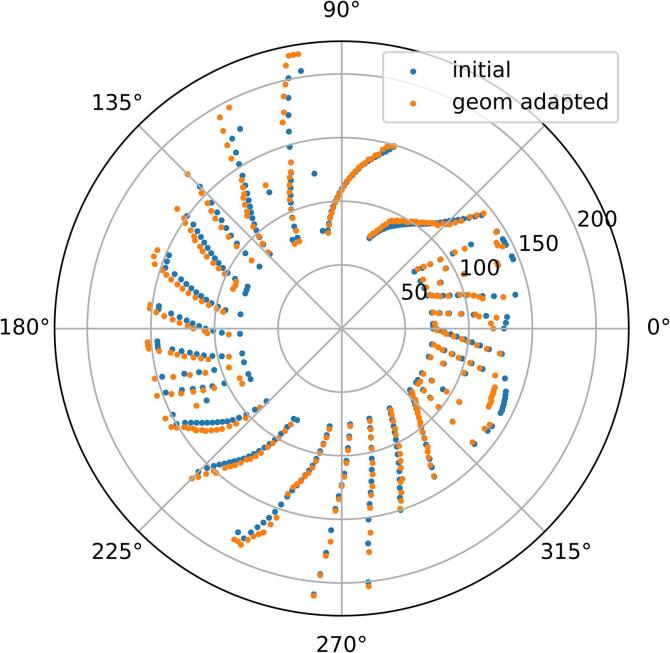
Example of energy layer pattern update. Radial position denotes the energy in MeV, angular position denotes the beam angle. “initial” = initial arc energy layer pattern on CT1; “geom adapted” = geometrically adapted energy layer pattern on CT2.

Fig. 2 shows an example of energy layer adaptation after workflow steps 1 and 2, demonstrating preserved angular positions of energy switch-ups. Table 1 indicates target coverage and SC maximum dose for all patients when using plan computed for CT_1_ on CT_2_ (No adaption) and when using the geometric plan adaption. For all cases the D_98%_ is noticeably below 66.5 Gy, i.e., 95 % of the prescription dose. Geometric adaption slightly improves the target coverage and SC maximum dose for some patients. However, it was not sufficient to recover clinical quality.

**Table 1:** Comparison between no adaptation, geometric adaptation (without spot weights adaptation) and spot weights adaptation (with geometric adaptation first) on CT_2_ for nominal case for D98% of CTVp, D_98%_ of CTV_n_ and the maximal dose received (D_max_) by spinal cord (SC) the 5 patients.

		P1	P2	P3	P4	P5
CTV_p_ D_98_ (Gy)	Clinical goal	>66.5 Gy				
	No adaptation	29.1	53.0	62.7	56.4	65.2
	Geometric	30.4	56.1	64.4	57.5	62.4
	Spot weights	68.2	67.9	68.0	68.4	68.3
CTV_n_ D_98_ (Gy)	Clinical goal	>51.54 Gy				
	No adaptation	24.9	39.4	40.9	45.8	47.7
	Geometric	30.4	41.7	41.5	44.0	42.1
	Spot weights	52.8	53.0	53.4	53.0	53.2
SC D_max_ (Gy)	Clinical goal	<44 Gy				
	No adaptation	66.6	41.9	30.3	22.6	24.7
	Geometric	62.1	36.4	29.3	24.9	24.1
	Spot weights	32.8	27.3	21.9	26.5	22.6

### 3.2 Spot weights adaptation

Table 1 also includes details for target coverage and SC maximum dose of the spot weights adaptation. The constraints on the targets and on the SC for the nominal case were largely met. Target coverage objectives and SC D_max_ objectives were also satisfied for the reference plans. Table 2 displays metric differences between plans that were adapted and their reference. We can notice a slight median decrease for D_98%_ of 0.21 Gy for CTV_p_ and 0.44 Gy for CTV_n_, while still being largely above the clinical constraint (i.e., 64.4 Gy and 51.54 Gy, respectively) for all plans. D_max_ difference for SC was globally a bit lower for the smart-adapted plans even if there is one smart-adapted case exhibiting an excess of 0.49 Gy compared to the reference plan. Mean dose differences to OARs were globally low: the highest median difference over all patients was found for the mandible and was of 1.86 Gy.

**Table 2:** Dose volume-metrics difference between smart-adapted plans and reference plans on CT_2_ for nominal case. SC: spinal cord.

Volume	Metric	Smart adaptation minus Reference	
		Median (Gy)	Range (Gy)
CTV_p_	D_98%_	−0.2	[-0.6, 0.1]
CTV_p_	D_2%_	0.2	[-0.1, 0.7]
CTV_n_	D_98%_	−0.4	[-0.5, −0.1]
CTV_n,high_	D_98%_	−0.4	/
CTV_n,high_	D_2%_	0.0	/
SC	D_max_	−0.5	[-5.0, 1.2]
SC	D_mean_	0.1	[-1.6, 0.8]
Mandible	D_mean_	1.9	[-8.7, 4.4]
Parotid_L	D_mean_	0.9	[-3.6, 1.9]
Parotid_R	D_mean_	0.1	[-0.8, 2.7]
Thyroid	D_mean_	0.6	[-2.7, 1.6]
Esophagus	D_mean_	−2.2	[-3.9, 2.1]
Larynx	D_mean_	−0.1	[-1.3, 2.5]
Oral Cavity	D_mean_	0.7	[-4.1, 1.2]

Concerning dose-volume differences for the worst-case scenario, results are displayed in Table 3. The trend is similar to the nominal case: a slight decrease in CTV_p_ and CTV_n_ coverage of 0.44 and 0.56 Gy, respectively; while CTV_p_ D_2%_ was 0.24 Gy higher, thus resulting in a lower dose homogeneity. As for the nominal case, D_max_ to SC was about 1.47 Gy lower for smart-adapted plans than for reference plans.

**Table 3:** Critical dose volume-metrics difference between smart-adapted plans and reference plans on CT_2_ for worst case. SC: spinal cord.

Volume	Metric	Smart adaptation minus Reference	
		Median (Gy)	Range [min, max] (Gy)
**CTV_p_**	D_98%_	−0.4	[-0.8, −0.2]
**CTV_p_**	D_2%_	0.2	[-0.1, 1.0]
**CTV_n_**	D_98%_	−0.6	[-0.7, 0.0]
**CTV_n,high_**	D_98%_	−0.3	/
**CTV_n_high_**	D_2%_	0.4	/
**SC**	D_max_	−1.5	[-3.1, 1.3]
**Body**	D_1cc_	0.7	[-0.4, 1.9]

### 3.3 Timing analysis

Section “Spots initialization” in Table 4 displays the timings of the different steps. During spot initialization (steps 1 and 2), our adaptive workflow was 7.7 s slower than the reference method of generating a new spot grid and optimizing energy layers with ELSA, resulting in a relative speed factor of 0.76. Section “Dose influence computation” in Table 4 reports computation times to generate the dose influence matrix (step 3). The proposed adaptative workflow was on average 3.55 faster than the reference one. Concerning the optimization part in the section “spots weight optimization” (step 4 of the adaptive workflow), the smart-adaptation constantly re-optimized less than 50 % of the total spots of CT_1_ onto CT_2_. This step only considers the required time to optimize the spot weights. The adaptation workflow was on average 2.32 times faster than the reference one. Overall, the adaptive workflow was 3.36 times faster than the reference method.

**Table 4:** Timing comparison between reference (Ref) and adaptation workflow (Smart) for the 5 patients. The “average acceleration of the step” rows are computed as average of reference divided by average of adaptation workflow. The “Average acceleration of plan adaption” row on the bottom is computed by dividing the sum of the time components of the reference workflow by the sum of the different time components of the smart-adaptive workflow. Percentage of re-optimized spots are also provided.

Step	Method	Details	Patient 1	Patient 2	Patient 3	Patient 4	Patient 5
Spots initialization	Ref (s)	Spot + energy layer determination	47	36	37	38	34
	Smart (s)	Registration determination Time	26	47	16	23	12
		Spots displacement and energy update	26	24	27	24	26
	Average acceleration of the step		0.76				
Dose influence computation	Ref (s)		15,523	28,538	15,371	6253	20,938
	Smart (s)		4975	6470	5083	2097	5001
	Average acceleration of the step		3.55				
Spots weight optimization	Ref (s)		2132	1308	1496	2817	1350
	Smart (s)		860	928	752	628	1080
	Average acceleration of the step		2.32				
	optimized spots (%)		46.6	46.8	47.4	48.0	49.5
Overall average acceleration of the whole adaptation process			3.36				

## 4 Discussion

We implemented a “smart” plan adaptation workflow for PAT and evaluated it on 5 head and neck cancer patients. The workflow features a geometric and a spot weight step. It is inspired by the work of Botas et al. for IMPT [25] while making some adaptation to accommodate it to PAT modality, as well as adding a novel criteria to select the most relevant spots for re-optimization.

Geometric adaptation showed modest improvements in target coverage for some patients and failed to achieve clinically acceptable quality. Target coverage, and sometimes the maximum dose to the SC, did not meet the clinical constraints. In some cases (patients 4 and 5) geometric adaptation seemed to be worse than no adaptation. This followed a similar trend observed in Botas' paper and highlighted the need for re-optimization of spot weights for complex cases. The limited success of geometric adaptation might stem from our patient selection, as these cases initially required replanning due to substantial anatomical changes. Such changes might have exceeded the capabilities of the geometric adaptation.

The spot weights adaptation step, which re-optimized less than 50 % of the total spots, produced treatment plans comparable to their reference counterparts created from the ground up. While the smart-adapted plans exhibited a minor reduction in dose homogeneity, this did not meaningfully impact their clinical acceptability.

Although marginally slower in initial spot and energy determination, the smart adaptation method achieved significant speedups in subsequent steps i.e., dose influence matrix computation and dose optimization. Smart adaptation method proved to be 3.55 times faster than classical optimization from the ground up (reference) for the computation of the dose influence matrix and 2.32 times faster for the dose optimization using the optimization constraint from CT_1_. Such acceleration for beamlets generation stems from simulating fewer protons by leveraging prior weight information. The dose optimization acceleration results from both the initialization of the spot weights that lead the optimization process to start from a better initial solution and the reduction of total number of spot weights to optimize thus resulting in a less computationally intensive process.

However, among various approaches for energy layer determination, some could be significantly slower than the ELSA method used here [12], [15]. These findings suggest the proposed method may have even greater impact when applied to these slower techniques by allowing quick updates to initial angle/energy patterns rather than re-launching a new determination process. Future tests should evaluate the time savings for these other energy layer algorithms.

An interesting perspective would be evaluating this workflow on commercial TPS where some operations are generally faster than in OpenTPS. Currently, the time span to produce a smart-adapted plan remains hardly compatible with clinical conditions. Notably, only a few matrix operations are GPU-implemented in this TPS, while in the original Botas’ paper, most workflow operations were GPU-implemented, resulting in a time-scale compatible with OA. Therefore, evaluating the proposed workflow on different systems, would be instructive. However, in contrary to OpenTPS, certain parameters tuned in this workflow may not be readily accessible within these commercial systems. For example, saving spot dose contributions and reusing them for subsequent calculations, as well as freezing optimization of discarded spot weights, could be challenging due to their lack of accessibility to users.

Regarding optimization constraints, weight updates to target coverage and maximum dose to the SC were necessary. This adjustment loop is not perfectly compatible with OA. In addition, reusing constraints from CT_1_ may theoretically be suboptimal for CT_2_. However, in recent years, several techniques of AI constraint prediction and automatic planning have proven their efficiency [30], [31], [32]. Combining such predictions with this adaptive workflow would likely be valuable. Future work should investigate compatibility with updated constraints that are more specific to the anatomy of the day, i.e., reduce dose to OARs on the CT of the day when possible by the use of stricter constraints.

In conclusion, while the proposed workflow achieved comparable dose-volume metrics to full re-optimization with substantial time savings, further development is needed to achieve adaptation times compatible with online adaptation.

## CRediT authorship contribution statement

**Benjamin Roberfroid:** Data curation, Formal analysis, Investigation, Methodology, Software, Visualization, Writing – original draft. **Margerie Huet-Dastarac:** Software, Writing – original draft. **Elena Borderías-Villarroel:** Data curation, Writing – original draft. **Rodin Koffeing:** Resources. **John A. Lee:** Conceptualization, Supervision, Writing – original draft. **Ana M. Barragán-Montero:** Conceptualization, Supervision, Writing – original draft. **Edmond Sterpin:** Conceptualization, Supervision, Writing – original draft, Funding acquisition.

## Declaration of competing interest

The authors declare the following financial interests/personal relationships which may be considered as potential competing interests: Benjamin Roberfroid and Margerie Huet Dastarac are supported by the 10.13039/100018985Walloon Region under grant agreement MECATECH / BIOWIN No. 8090 (Proton Arc Therapy).
