# Supplementary material for: Towards faster plan adaptation for proton arc therapy using initial treatment plan information
Source: Phys Imaging Radiat Oncol. 2025 Jan 30;33:100705. doi: 10.1016/j.phro.2025.100705 (PMC11851183; doi:10.1016/j.phro.2025.100705)
Supplement: Supplementary Data 1 [file mmc1.pdf]

Supplementary materials

Volume	Metric	Smart adaptation minus Reference	
		Median (Gy)	Range [min, max](Gy)
**CTV_p**	$D_{98\%}$	-0.4	[-0.8, -0.2]
**CTV_p**	$D_{2\%}$	0.2	[-0.1, 1.0]
**CTV_n**	$D_{98\%}$	-0.6	[-0.7, 0.00]
**$CTV_{n,high}$**	$D_{98\%}$	-0.3	/
**CTV_{n_high}**	$D_{2\%}$	0.4	/
**SC**	D_{max}	-1.5	[-3.1, 1.3]
**Body**	D_{1cc}	0.7	[-0.4, 1.9]
**Mandible**	D_{mean}	1.6	[-8.1, 4.1]
**Parotid Left**	D_{mean}	1.2	[-1.7, 1.9]
**Parotid Right**	D_{mean}	0.2	[-0.6, 2.3]
**Thyroid**	D_{mean}	-0.3	[-3.0, 1.5]
**Esophagus**	D_{mean}	-2.0	[-4.7, 3.7]
**Larynx**	D_{mean}	0.6	[-0.5, 2.4]
**Oral cavity**	D_{mean}	0.3	[-3.6, 1.3]

Table A1: Extension of table 3 (worst-case) with extra values for the Dmean of OARs volume. Critical dose volume-metrics difference between smart-adapted plans and reference plans on CT2 for worst case. SC: spinal cord.

Table A2: Spot selection according to the different criteria. First column (S1) refers to the selection of the smallest subset of spots carrying at least 65% of the total spot weight. Second column refers to the spot selection when adding the second criterion (S2, $\frac{\partial F}{\partial w_i} > t$) to S1. Third column refers to the spots that would be selected if the same criterion than reference paper from Botas et al. would have been chosen (OC), i.e. the smallest subset of spots carrying at least 50% of the total weight.

	% spots kept after selection of initial weight (S1)	% spots kept after S1 and objective function gradient constraint (S1+S2)	% spots selection *Botas et al.* criterion (OC)
P1	55.6	46.6	39.8
P2	57.5	46.8	42.1
P3	53.6	47.4	38.1
P4	50.7	48.0	35.5
P5	55.2	49.5	39.7

Table A3: Extension of table 2 with addition of dose-volume values achieved with the OC criterion for nominal case. SC: spinal cord.

**Volume**	**Metric**	Smart adaptation minus Reference		OC adaptation minus Reference	
		**Median (Gy)**	**Range (Gy)**	**Median**	**Range [min, max] (Gy)**
**CTV_p**	$D_{98\%}$	-0.2	[-0.6, 0.1]	-0.2	[-0.7, 0.10]
**CTV_p**	$D_{2\%}$	0.2	[-0.1, 0.7]	0.1	[0.0, 0.8]
**CTV_n**	$D_{98\%}$	-0.4	[-0.5, -0.1]	-0.5	[-0.6, -0.3]
**$CTV_{n,high}$**	$D_{98\%}$	-0.4	/	-0.2	/
**$CTV_{n,high}$**	$D_{2\%}$	-0.0	/	0.0	/
**SC**	D_{max}	-0.5	[-5.0, 1.2]	-0.5	[-4.8, 2.2]
**SC**	D_{mean}	0.1	[-1.6, 0.8]	0.1	[-0.5,1.9]
**Mandible**	D_{mean}	1.9	[-8.7, 4.4]	1.9	[-8.8,4.6]
**Parotid_L**	D_{mean}	0.9	[-3.6, 1.9]	0.9	[-3.5, 2.3]
**Parotid_R**	D_{mean}	0.1	[-0.8, 2.8]	0.0	[-1.9, 2.1]
**Thyroid**	D_{mean}	0.6	[-2.7, 1.7]	0.0	[-2.7, 1.2]
**Esophagus**	D_{mean}	-2.2	[-3.9, 2.1]	2.3	[-3.9, 1.4]
**Larynx**	D_{mean}	-0.1	[-1.3, 2.5]	0.0	[-1.3, 3.4]
**Oral Cavity**	D_{mean}	0.7	[-4.1, 1.2]	0.7	[-4.0, 1.1]

Table A4: Extension of table 3 with addition of dose-volume values achieved with the OC criterion for worst case. SC : spinal cord. OC consistently provided a less homogeneous target coverage, a higher D_{max} to SC and D1cc to body. Such differences were sometimes large enough to fall below expected minimal target coverage i.e >66.5Gy for CTV_p and >51.54Gy for CTV_n, while it was not the case for the S1+S2 selection. Using OC, CTV_p coverage 1/5 patient met the target coverage goal, for CTV_n 2/5 and for $CTV_{n,high}$ 0/1.

Volume	Metric	Smart adaptation minus Reference		OC adaptation minus Reference	
		**Median (Gy)**	**Range [min, max] (Gy)**	**Median (Gy)**	**Range [min, max] (Gy)**
**CTV_p**	$D_{98\%}$	-0.4	[-0.8, -0.2]	-0.62	[-0.9, -0.3]
**CTV_p**	$D_{2\%}$	0.2	[-0.1, 1.0]	0.49	[-0.2, 1.3]
**CTV_n**	$D_{98\%}$	-0.6	[-0.7, 0.0]	-0.66	[-1.1, -0.3]
**$CTV_{n,high}$**	$D_{98\%}$	-0.3	/	-0.49	/
**CTV_{n_high}**	$D_{2\%}$	0.4	/	0.49	/
**SC**	D_{max}	-1.5	[-3.1, 1.3]	-0.95	[-1.9, 1.9]
**Body**	D_{1cc}	0.7	[-0.4, 1.9]	0.73	[-0.3, 2.1]
